# A Comparative Study Between Plate Osteosynthesis and Intramedullary Nailing for Diaphyseal Fracture of Radius and Ulna in Adults

**DOI:** 10.7759/cureus.37277

**Published:** 2023-04-08

**Authors:** Ramavtar Saini, Anshu Sharma, Kuldeep Baisoya, Divyaraj Ravalji

**Affiliations:** 1 Orthopedics, Geetanjali Medical College and Hospital, Udaipur, IND

**Keywords:** intramedullary nailing, modified grace eversmann scoring system, plate osteosynthesis, diaphyseal ulna fracture, diaphyseal radius fracture

## Abstract

Introduction: In this era of active living, industrial growth, increasing automobile accidents, and athletic activities, fractures of the forearm bones are becoming more frequent. The incidence of diaphyseal fractures of both bone forearms is reported to be approximately 10 per 10,000 persons per year, although rates may vary according to age and sex. If not properly treated, a fracture of the forearm bones might cause a serious loss of function. Therefore, to restore function, these fractures require adequate anatomical reduction and internal fixation. The majority of forearm fractures in adults are treated surgically, and various modes of internal fixation are available. In this study, we evaluated and compared the clinical, functional, and radiological outcomes of both bone forearm diaphyseal fractures treated with plate osteosynthesis and intramedullary nailing.

Material and method: This prospective and comparative study was conducted in a tertiary care medical teaching hospital in southern Rajasthan, India. Forty patients with diaphyseal fractures of the radius and ulna bones who presented to the casualty or orthopedic outpatient departments of our institute were included. Patients were divided into two groups, 20 patients in each group and treated by intramedullary nailing (group A) and plate osteosynthesis (group B), and regularly followed up and evaluated for clinical, functional, and radiological outcomes.

Result: Final results were calculated according to the modified Grace-Eversmann scoring system. In group A, out of 20 cases excellent score was seen in six cases (30%), good score in nine cases (45%), fair score in three cases (15%), and at last, two cases (10%) showed poor results. In group B, excellent score was in eight cases (40%), good score was in eight cases (40%), fair score was in three cases (15%), and at last, one case (5%) was poor in our study.

Conclusion: Based on our findings, we conclude that for the treatment of diaphyseal fractures of the radius and ulna, both treatment modalities provide equally satisfactory results.

## Introduction

Fractures of the radius and ulna are among the most common upper extremity fractures in adults [1]. The incidence is reported to be approximately 10 per 10,000 persons per year, although rates may vary according to age and sex [2]. Studies show a bi-modal distribution, with the highest incidence among young males aged 10 to 20 years (10:10,000) and females over age 60 years (5:10,000) [3,4].

The majority of adult forearm fractures are typically treated with conventional bone setters. The conservative treatment of forearm fracture is fraught with complications of casting, compartment syndrome, malunion, and non-union [5]. To stop this practice, it is imperative that surgeons are aware of the various options of surgical fixation and how they contribute to the effective management of forearm fractures [6].

Diaphyseal fractures of the radius and ulna are classified as articular fractures, so a slight deviation in the spatial orientation of both the radius and ulna minimizes the rotational frequency of the forearm, impairing hand positioning and functions. The preservation of inter-osseous space becomes necessary while treating fractures of the radius and ulna because it is necessary for successful pronation and supination to take place [5]. Therefore, to restore forearm functions, these fractures require adequate anatomical reduction and internal fixation [7].

Nowadays, the majority of forearm bone fractures in adults are treated surgically, and various modes of internal fixation are available, the choice of which is determined by the treating surgeon [8]. For diaphyseal fractures of forearm bones, open reduction and internal fixation (ORIF) with a dynamic compression plate (DCP) is a common procedure [9]. Intramedullary nailing is another vital option for forearm bone fracture fixation. Regardless of the implants used, the goal is to obtain union with an excellent functional outcome and early mobilization. In this study, we evaluated and compared the clinical, functional, and radiological outcomes of both bone forearm fractures treated either with plate osteosynthesis or nailing in our institute.

## Materials and methods

This randomized, prospective, and comparative study was conducted at a tertiary care medical teaching institute in southern Rajasthan, India. Ethical committee clearance was obtained (Institutional Ethics Committee, Geetanjali Medical College and Hospital, Udaipur, Rajasthan: GU/HREC/EC/2021/1928).

On presentation, patients were screened for other system injuries since they are closely associated with high-velocity trauma. Any associated neurovascular deficit has been ruled out at this stage and taken note of the same. Gentle manipulation is done to bring gross reduction of the fracture segment and an above-elbow support slab is applied. Patients meeting the requirements of inclusion and exclusion criteria were enrolled in this study on an inpatient basis after taking informed and written consent. A detailed history was obtained, and a thorough general examination was done with special emphasis on diaphyseal fractures of the radius and ulna. True anteroposterior and lateral radiographs were taken. All the fractures were classified using the Orthopedic Trauma Association (OTA) classification [10].

The inclusion criteria were: (1) age more than or equal to 18 years, (2) primary diaphyseal fracture of the radius and ulna bones, and (3) all closed or open fractures up to Gustilo-Anderson grade II [11]. Exclusion criteria were: (1) skeletally immature patient, (2) very narrow intramedullary canal, (3) pathological fractures, (4) single bone fractures, (5) presence of neurovascular deficit, (6) open fracture with Gustilo-Anderson grade III, and (7) patient with head injuries.

Forty patients were enrolled in this study after successfully meeting the inclusion and exclusion criteria and were randomly divided into two groups A and B with 20 patients in each group. Group A patients underwent internal fixation with intramedullary nailing, and in group B, patients underwent fixation with plate osteosynthesis. All relevant lab investigations, including coagulation studies, were carried out, and a pre-anesthetic checkup (PAC) was done. If the patient's medical status is in doubt, a medical team consultation is conducted. Patients were counseled about the outcome of end-functional results.

Surgical technique for plate osteosynthesis: Volar Henry’s approach [12] was used for radius plating, and a subcutaneous approach was used for ulna plating. Both fractures were exposed and reduced before fixation of either, fracture having less comminution was fixed first. Implants used for fixation were small fragment dynamic compression plates (DCPs) and 3.5 mm cortical screws.

Surgical technique for intramedullary nailing [13]: the patient was laid supine on the operation table with the affected limb on the radiolucent arm board. Nailing was performed under intraoperative image intensifier (c-arm) guidance. For ulnar nailing, a 1 cm longitudinal incision was made over the tip of the olecranon, and the triceps insertion was incised. Using a straight awl, an entry portal was made over the olecranon. No reaming was done. Under imaging control, a nail of the proper size was inserted through the olecranon and moved over the fracture site. Fracture reduction was achieved by traction and manipulation and confirmed under an image intensifier. The nail progressed into the distal fragment and was left only 5 mm outside the bone ends at the entry site.

For radius nailing two entrance locations are reported, immediately lateral to the Lister tubercle and from the radial styloid process. The wrist joint is 5 mm away from each of these entrance locations. After giving a stab incision, the entry portal was made with a straight awl directly in line with the medullary canal. Pre-bending of the nail was done to approximate the bow of the radius. The rest of the technique was the same as that used for the ulnar nailing.

The operated limb was immobilized in an above-elbow slab, and post-operative radiographs were obtained. The operated limb is kept in an elevated position until the edema in the fingers subsides. The dressing was done on the third and fifth post-operative days for both groups. Depending on the stability of the fixation, the upper limb was immobilized. Regular follow-up was advised at intervals of two weeks, six weeks, three months, and six months. And x-rays were done at every follow-up, barring the first one. Patients were assessed for pain, function, and complications at each follow-up, and functional outcome was evaluated according to the modified Grace-Eversmann score [14].

## Results

This study embraces a total of 40 cases of diaphyseal fracture of forearm bones in adults, which were divided into two groups, group A and group B (20 each). Post-operatively, all patients of both groups were followed regularly for a minimum period of six months, and clinical, radiological, and functional data were collected and documented.

In our study, we found that the mean age in the nailing group was 36.6 years and in the plating group was 40.9 years, with a range from 20 to 70 years. Maximum fracture incidence was seen in both groups between the ages of 31 and 50 years. The male patients were more prevalent in both groups; in group A, 16 cases (80%), and in group B, 18 cases (90%) were male. The prevalence is higher in men because they participate in more outside activities. The right side was common in both groups. In group A, 12 cases (60%) had right-side forearm fractures, while eight cases (40%) had left-side involvement. Similarly, in group B, 13 cases (65%) had right-forearm fractures, while seven cases (35%) had left-side forearm fractures. Both groups were statistically similar in terms of demographic variables (Table 1).

**Table 1:** Demographic variables of cases. The demographic variables of cases like age, gender, and side involvement in both groups were comparable, and no statistically significant difference was observed.

Variables	Group A (nailing)	Group B (plating)	P-value
Age (in years)	36.60 ± 13.22	40.90 ± 12.23	p>0.005
Gender	16 Males	18 Males	p>0.005
	4 Females	2 Females	
Side involvement	12 Right	13 Right	p>0.005
	8 Left	7 Left	

In both groups, the most common mode of injury was a road traffic accident (RTA). In intramedullary nailing group A, out of 20 cases, RTA was the cause of injury in nine cases (45%), fall from height was in three cases (15%), assault was in four cases (20%), and fall at home was in four cases (20%). In plating group B, the cause of injury was RTA in 10 cases (50%), fall from height was in four cases (20%), assault was in five cases (25%), and fall at home was in one case (5%) out of all 20 cases. Both groups were similar statistically, according to the mode of injury. According to the OTA classification, out of 20 cases, the intramedullary nailing group had seven (35%) patients of 22A2, one case (5%) of 22B3, five cases (25%) of 22C2, and seven cases (35%) of 22C3, while in the plating group, out of 20 cases, 22A3 was in five cases (25%), 22B3 in eight cases (40%), 22C1 in one case (5%), 22C2 in two cases (10%), and lastly 22C3 in four (20%). In the nailing group (group A), five patients (25%) were operated on within two days of injury, and the remaining 15 patients (75%) were operated on between the third and fifth day after trauma. In the plating group (group B), six patients (30%) were operated on within two days of trauma, and the remaining 14 patients (70%) were operated on between the third and fifth day after trauma (Table 2).

**Table 2:** MOI and fracture-related variables. MOI and fracture-related variables in both groups were comparable, and no statistically significant difference was observed. MOI: mechanism of injury, AO: Association of Osteosynthesis, OTA: Orthopedic Trauma Association.

Variable	Group A (nailing)	Group B (plating)	P-value
Mechanism of injury (MOI)			
Road traffic accident (RTA)	9 (45%)	10 (50%)	p>0.005
Assault	3 (15%)	4 (20%)	
Fall from height	4 (20%)	5 (25%)	
Fall at home	4 (20%)	1 (5%)	
Classification (AO/OTA classification)			
22A3	7 (35%)	5 (25%)	p>0.005
22B3	1 (5%)	8 (40%)	
22C1	0 (0%)	1 (5%)	
22C2	5 (25%)	2 (10%)	
22C3	7 (35%)	4 (20%)	

In this study, in the intramedullary nailing group (group A), the average surgical time was 57.25 minutes, with a range of 35 to 75 minutes. In the plating group (group B), the average surgical time was 75.50 minutes, with a range of 56 to 90 minutes. In our study, in group A (nailing), the mean hospital stay was 4.05 days, whereas in group B (plating), the mean hospital stay was 6.35 days.

In both groups, the majority of fractures were successfully united. In group A (nailing), out of 20 cases, 18 cases (90%) had union within 14 weeks after surgery, one case (5%) had delayed union, which united by the 25th week, and only one patient showed non-union. In group B (plating), 19 cases (95%) had union within 15 weeks post-surgery, and one case (5%) had delayed union and eventually united by the 20th week after surgery. The average union time in group A was 10.43 weeks, whereas in group B it was 13.4 weeks.

In the nailing group, after surgical fixation, out of 20 cases, eight patients (40%) regained elbow flexion range more than 120°, six patients (30%) had a range between 100° and 120°, five cases (25%) had a range between 80° and 100°, and one case (5%) had a flexion range less than 80°. Whereas in the plating group, eight cases (40%) had an elbow flexion more than 120°, 10 cases (50%) had a flexion range of 100°-120°, and two cases (10%) had a flexion range of 80°-100°.

At the final follow-up, in group A (nailing), 11 patients (55%) had a pronation range of 60°-80°, eight cases (40%) had a range of 40°-60°, and only one case (5%) had a pronation range less than 40°, whereas in group B (plating), three cases (15%) had a pronation range of more than 80°, 12 patients (60%) had a range of 60°-80°, and five cases (25%) had a range of 40°-60°.

After surgical fixation at the final follow-up in the intramedullary nailing group (group A), seven cases (35%) had a supination range of more than 80°, seven patients (35%) had a range of 60°-80°, and six cases (30%) had a range of 40°-60°. Whereas in the plating group (group B), 10 cases (50%) had a supination range of more than 80°, six cases (30%) had a range of 60°-80°, and four patients (20%) had a range of 40°-60° (Table 3).

**Table 3:** Intraoperative and post-operative variables.

Variable	Group A (nailing)	Group B (plating)	p-value
Mean surgery time (in minutes)	57.25	75.5	p<0.005
Mean hospital stay (in days)	4.05	6.35	p<0.005
Union time (in weeks)	10.43	13.4	p<0.005
Range of motion			
Average supination	72°	76.75°	p>0.005
Average pronation	65.25°	69.5°	p>0.005
Average elbow flexion range	111.5°	118.42°	p<0.005

Post-operatively, on regular follow-up for a minimum of six months, we observed that in the intramedullary nailing group (group A), out of 20 cases, surgical site infection (SSI) occurred in two cases (10%), nail impingement was seen in three cases (15%), olecranon bursitis in three cases (15%), and one case (5%) showed delayed union and non-union. In the plate osteosynthesis group (group B), out of 20 cases, SSI occurred in three cases (15%), elbow stiffness in three cases (15%), delayed union in one case, and 13 (65%) cases have reported no complication (Table 4).

**Table 4:** Complications in the distribution between two groups.

Complications	Group A (nailing)	Group B (plating)
Surgical site infection (SSI)	2 (10%)	3 (15%)
Nail impingement	3 (15%)	0 (0%)
Olecranon bursitis	3 (15%)	0 (0%)
Elbow stiffness	0 (0%)	3 (15%)
Delayed union	1 (5%)	1 (5%)
Non-union	1 (5%)	0 (0%)
No complication	10 (50%)	13 (65%)

In our study, the final functional outcome was calculated according to the modified Grace-Eversmann scoring system. In the nailing group (group A), out of 20 cases, excellent score was seen in six cases (30%), good score in nine cases (45%), fair score in three cases (15%), and at last, two cases (10%) showed poor results. In the plating group (group B), excellent scores were observed in eight cases (40%), good scores in eight cases (40%), fair scores in three cases (15%), and at last, one case (5%), had a poor outcome out of 20 cases (Table 5).

**Table 5:** Final outcome distribution between two groups according to the modified Grace-Eversmann scoring system.

Final score range	Group (A) nailing	Percentage (%)	Group (B) plating	Percentage (%)
Excellent	6	30%	8	40%
Good	9	45%	8	40%
Fair	3	15%	3	15%
Poor	2	10%	1	5%
Mean	8		8.5	
Standard deviation (SD)	1.87		1.59	
P-value	p=0.2014			

**Figure 1:**
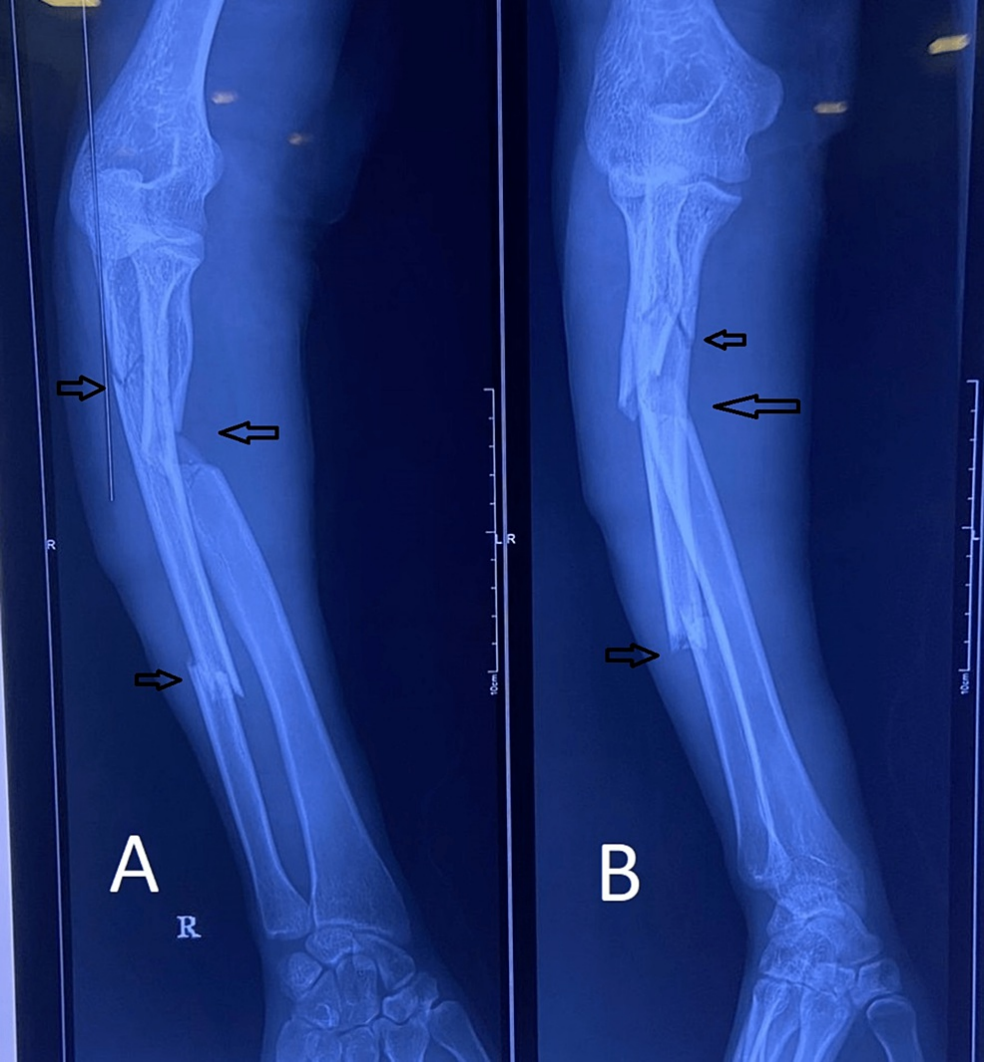
Pre-operative radiograph showing diaphyseal fracture of the ulna and radius. Pre-operative radiograph showing diaphyseal fractures of the ulna and radius bones (marked with arrows). Figure A is the anteroposterior (AP) view, and figure B is the lateral view.

**Figure 2:**
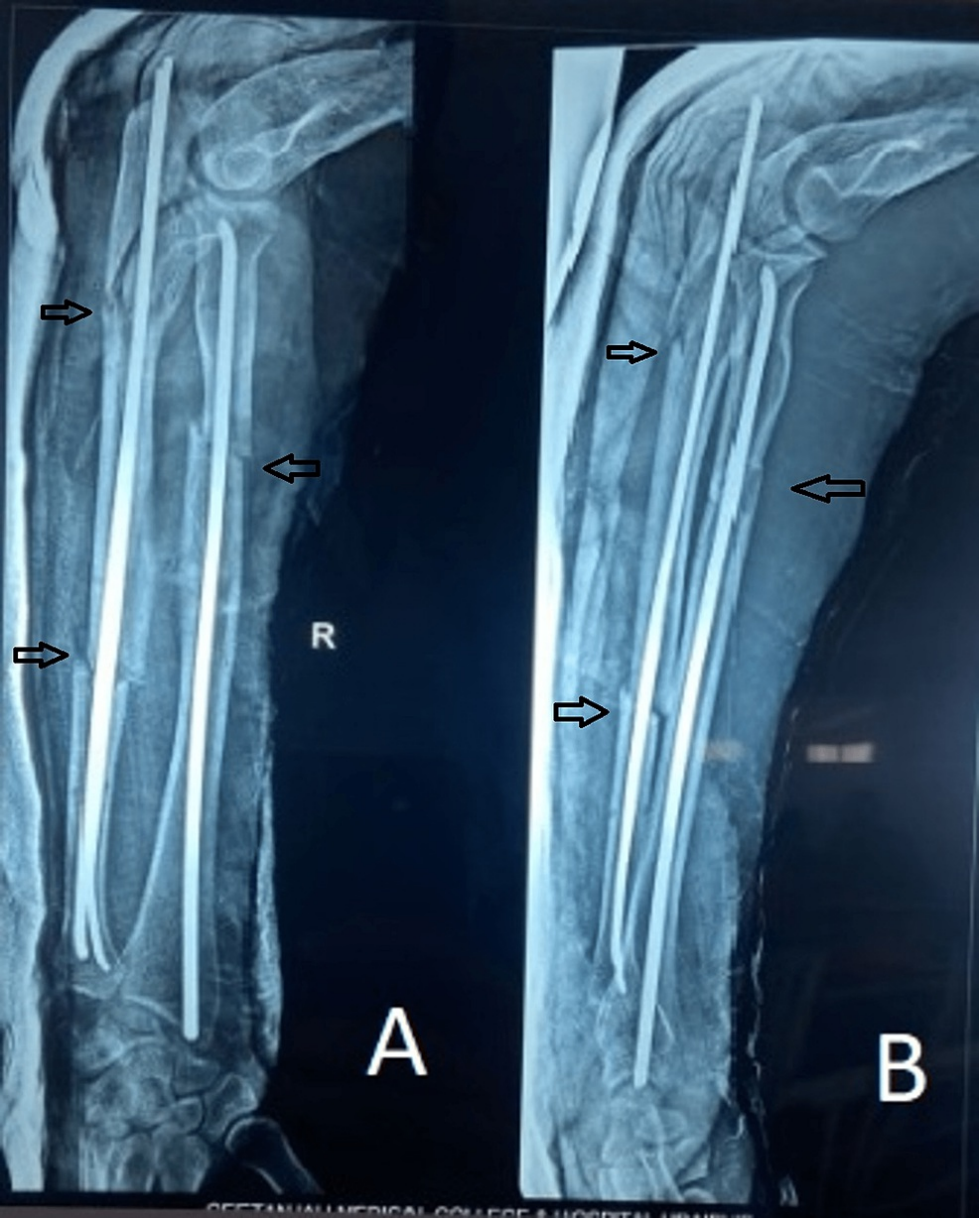
Post-operative radiograph. Post-operative radiograph showing fixation of the fracture (marked with arrows) with intramedullary titanium elastic nails. Figure A is the anteroposterior (AP) view, and figure B is the lateral view.

**Figure 3:**
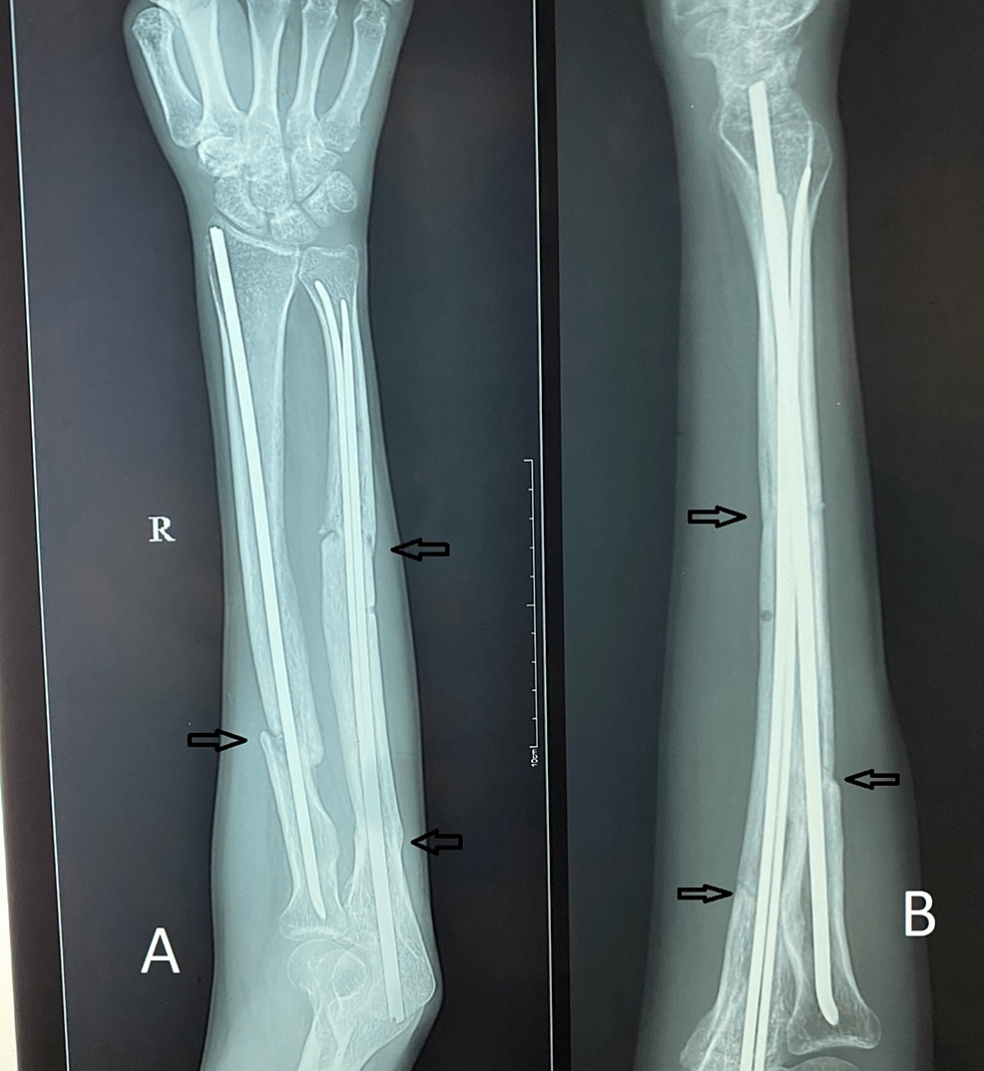
Three-month post-operative follow-up radiograph. Three-month follow-up radiograph showing progressive fracture union (marked with arrows). Figure A is the anteroposterior (AP) view, and figure B is the lateral view.

**Figure 4:**
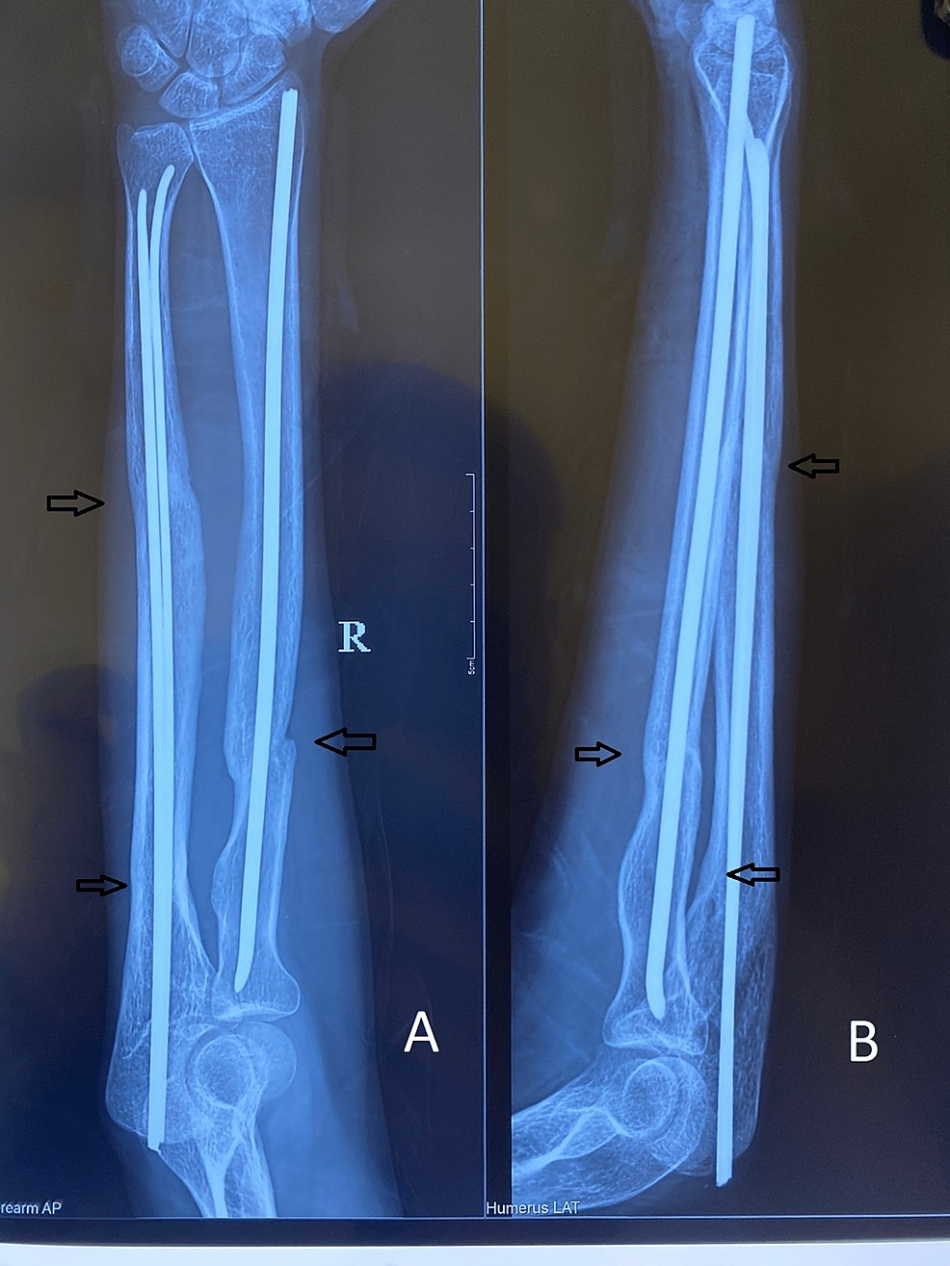
Sixth-month post-operative follow-up radiograph. Final follow-up radiograph showing fracture union (marked with arrows). Figure A is the anteroposterior (AP) view, and figure B is the lateral view.

**Figure 5:**
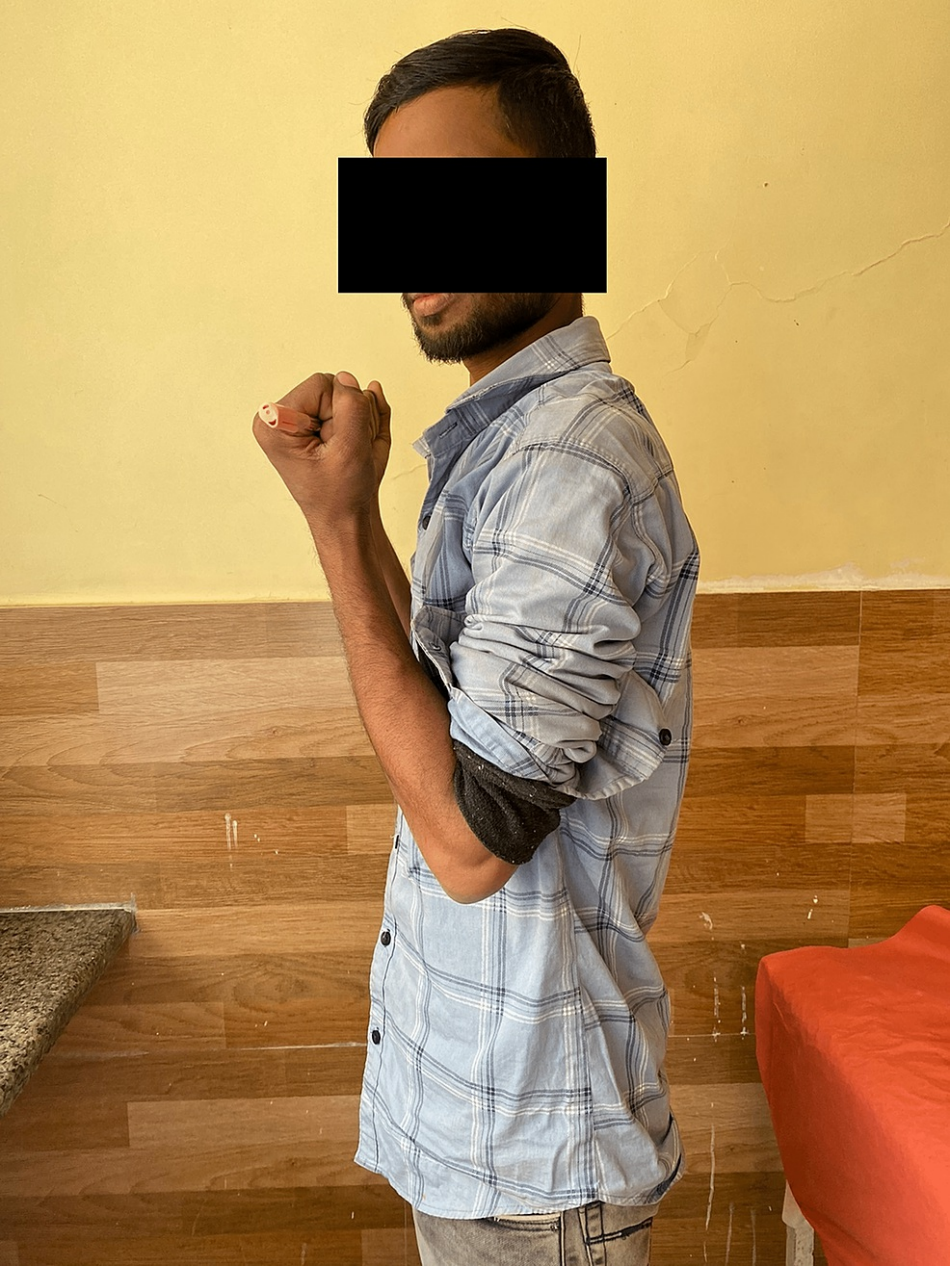
Clinical photograph at the final follow-up showing flexion movement at the elbow.

**Figure 6:**
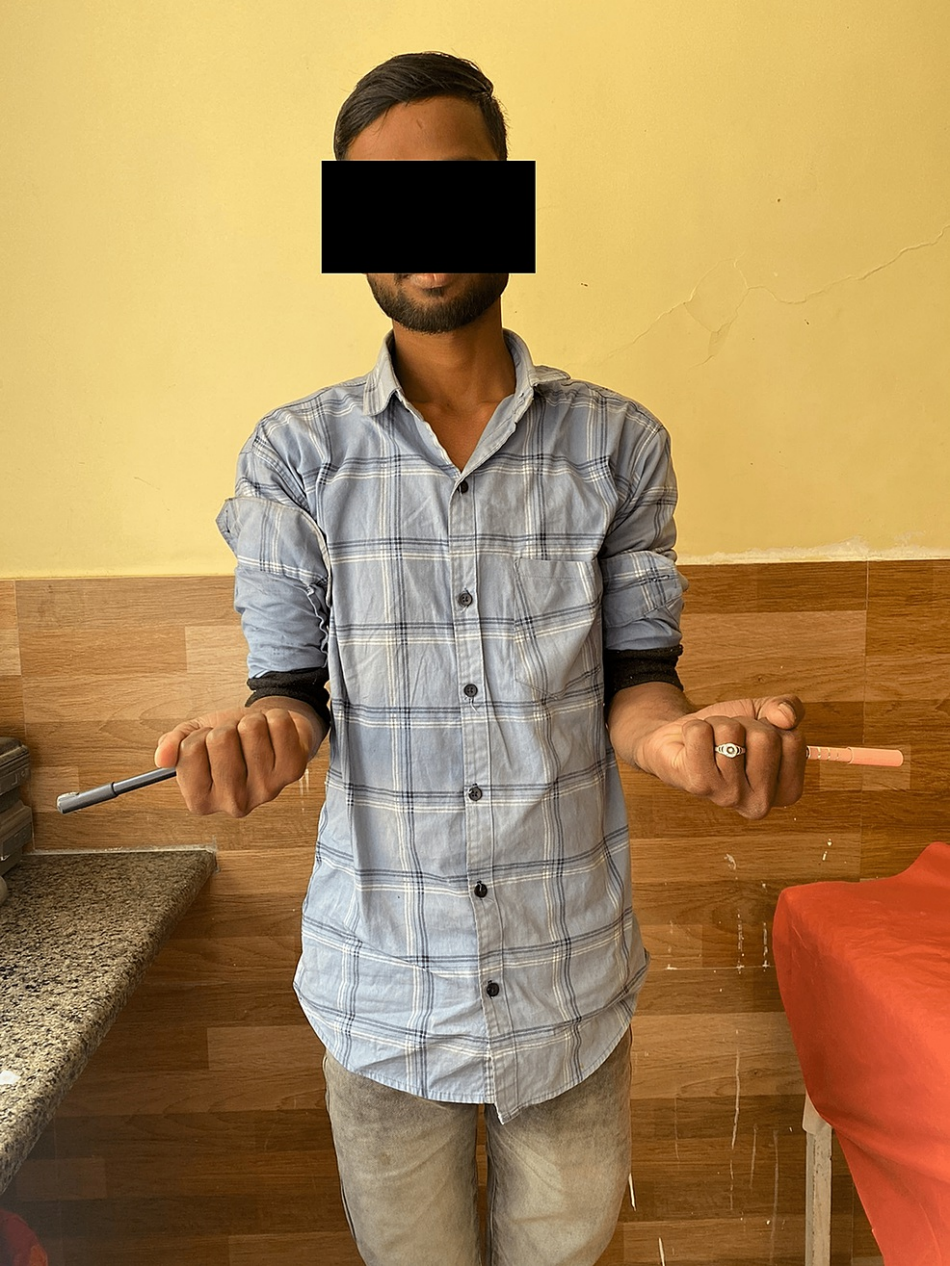
Clinical photograph showing supination movement at the elbow joint.

**Figure 7:**
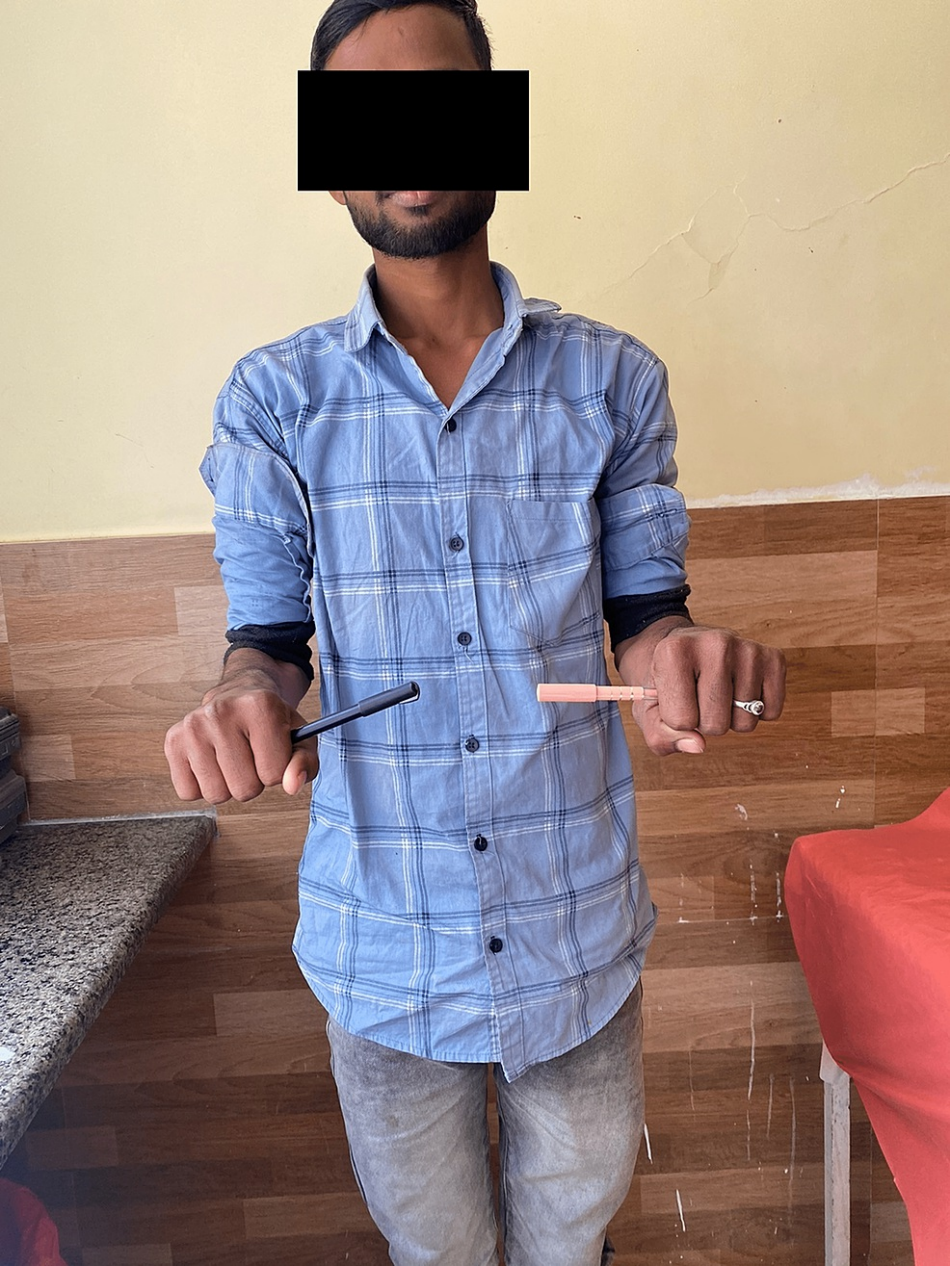
Clinical photograph showing pronation movement at the elbow joint.

Following are the radiographic images of one of our study cases managed by closed reduction and internal fixation with intramedullary nails (Figures 1, 2, 3, 4) and clinical images at the final follow-up (Figures 5, 6, 7).

**Figure 8:**
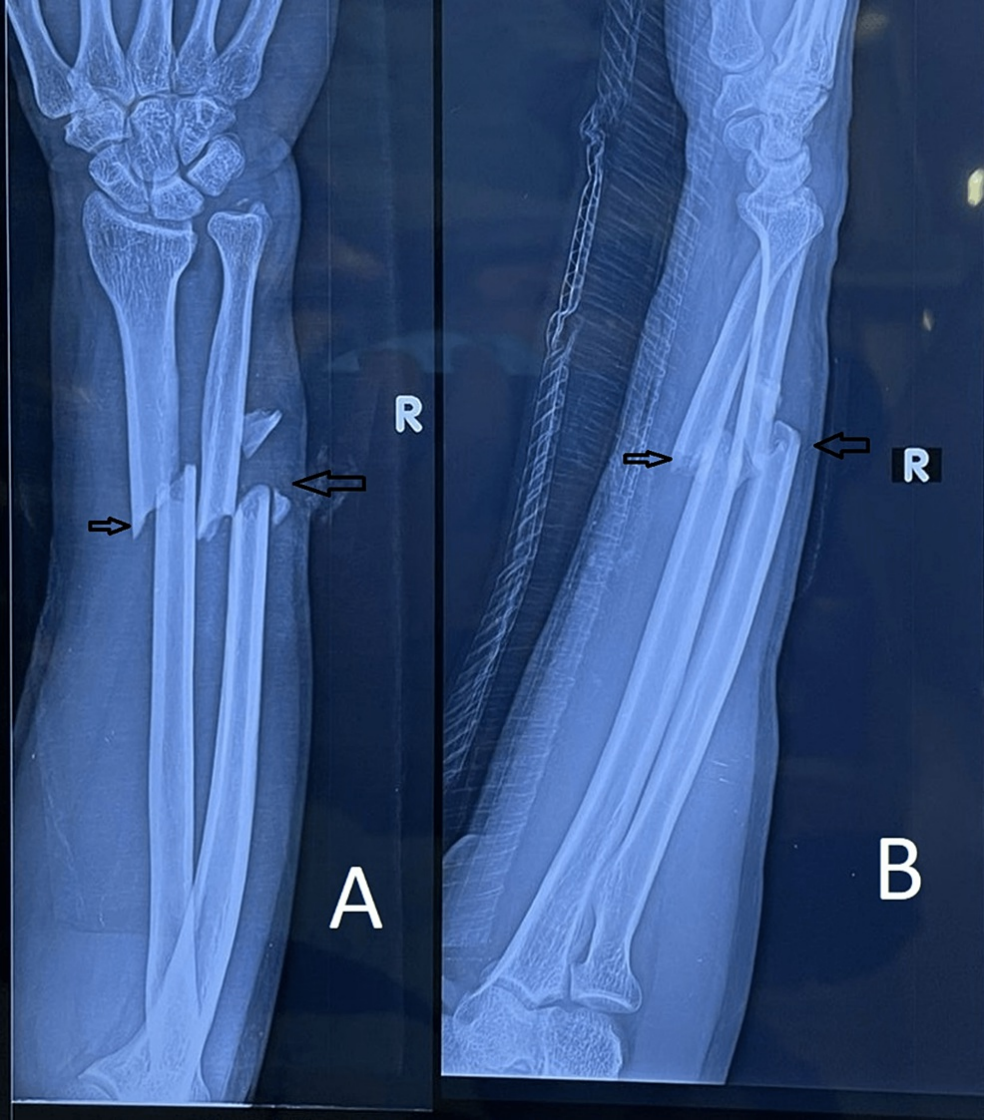
Pre-operative radiograph showing diaphyseal fracture of the ulna and radius. Figure A is the anteroposterior (AP), and figure B is the lateral view.

**Figure 9:**
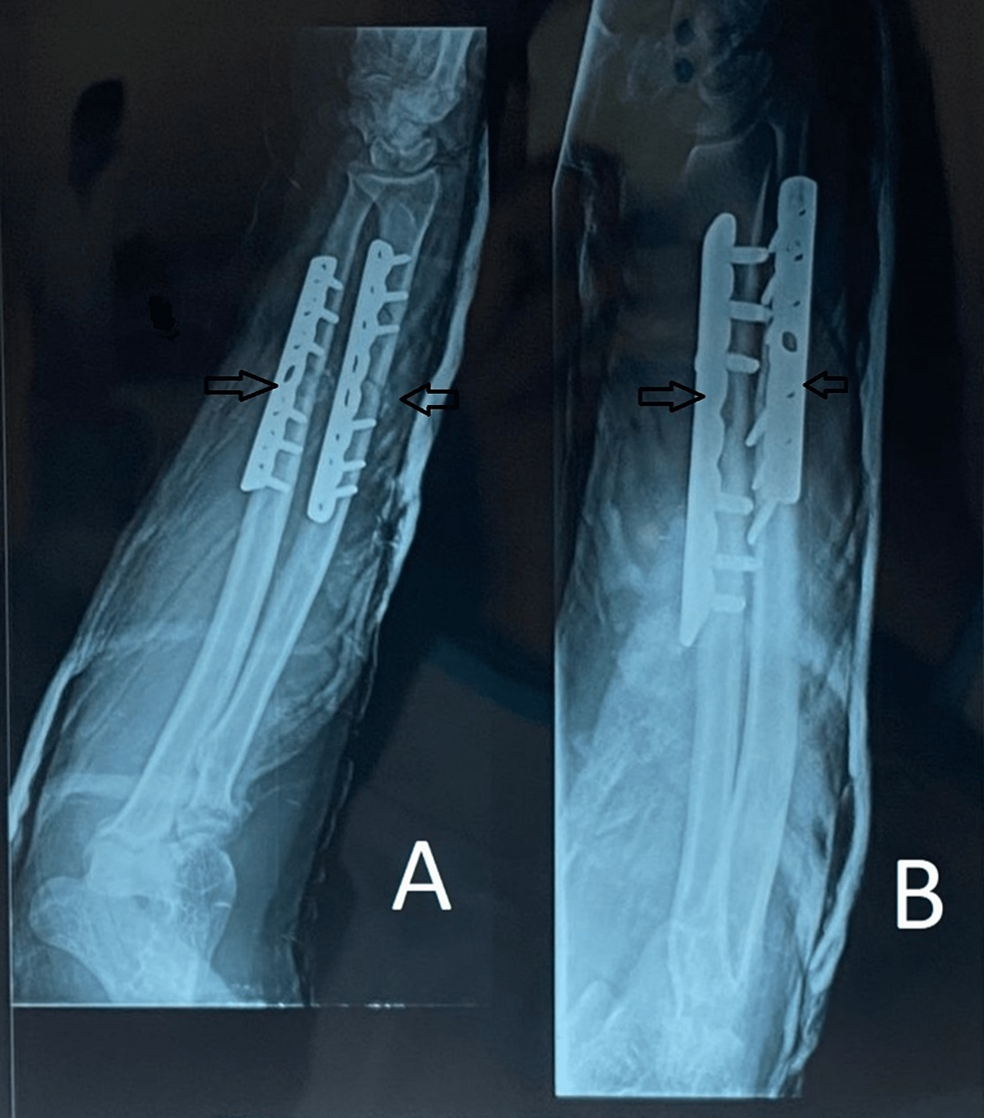
Post-operative radiograph. Post-operative radiograph showing fixation of the fracture (marked with arrows) with small dynamic compression plates (DCPs). Figure A is the anteroposterior (AP), and figure B is the lateral view.

**Figure 10:**
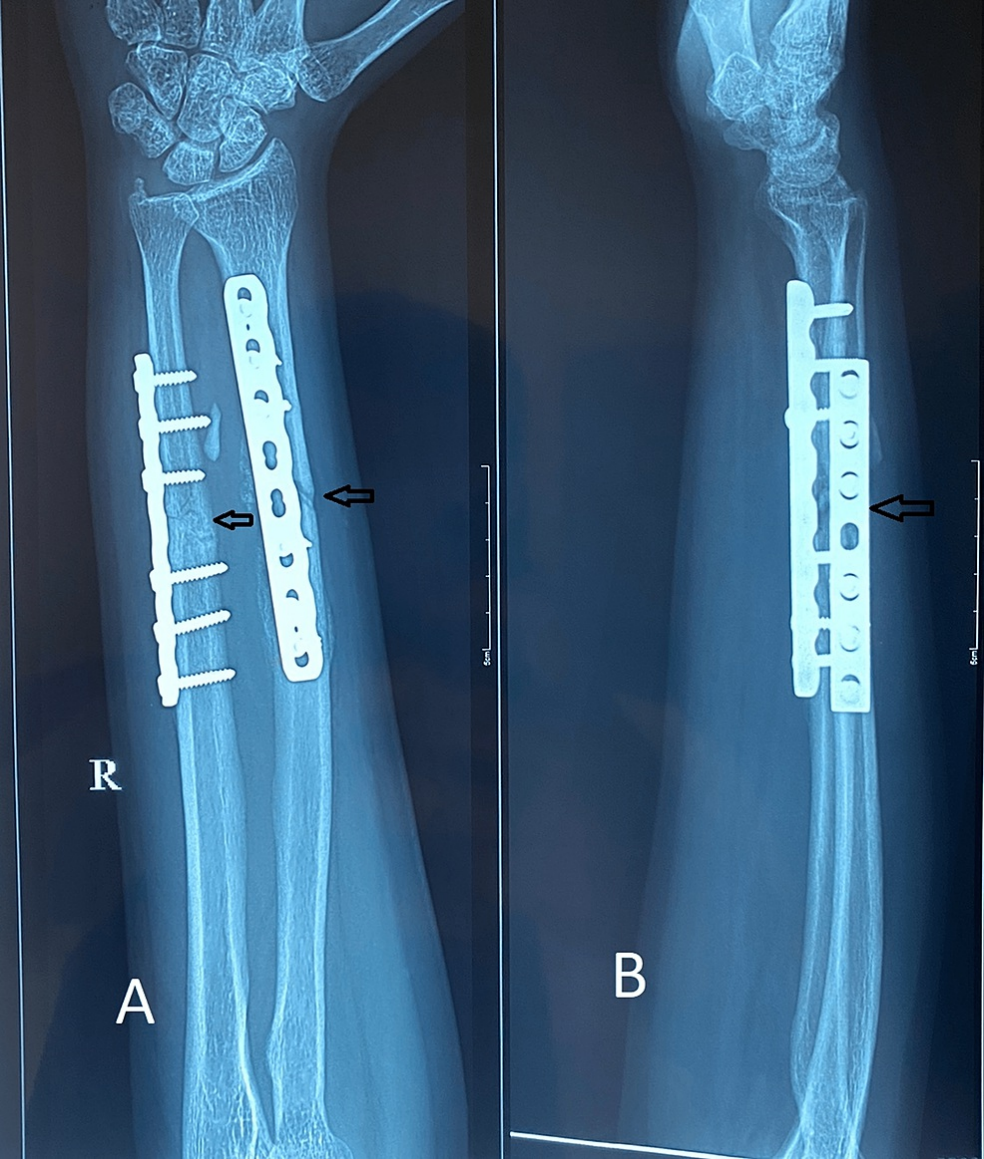
Three-month post-operative follow-up radiograph. Three-month follow-up radiograph showing progressive fracture union (marked with arrows). Figure A is the anteroposterior (AP) view, and figure B is the lateral view.

**Figure 11:**
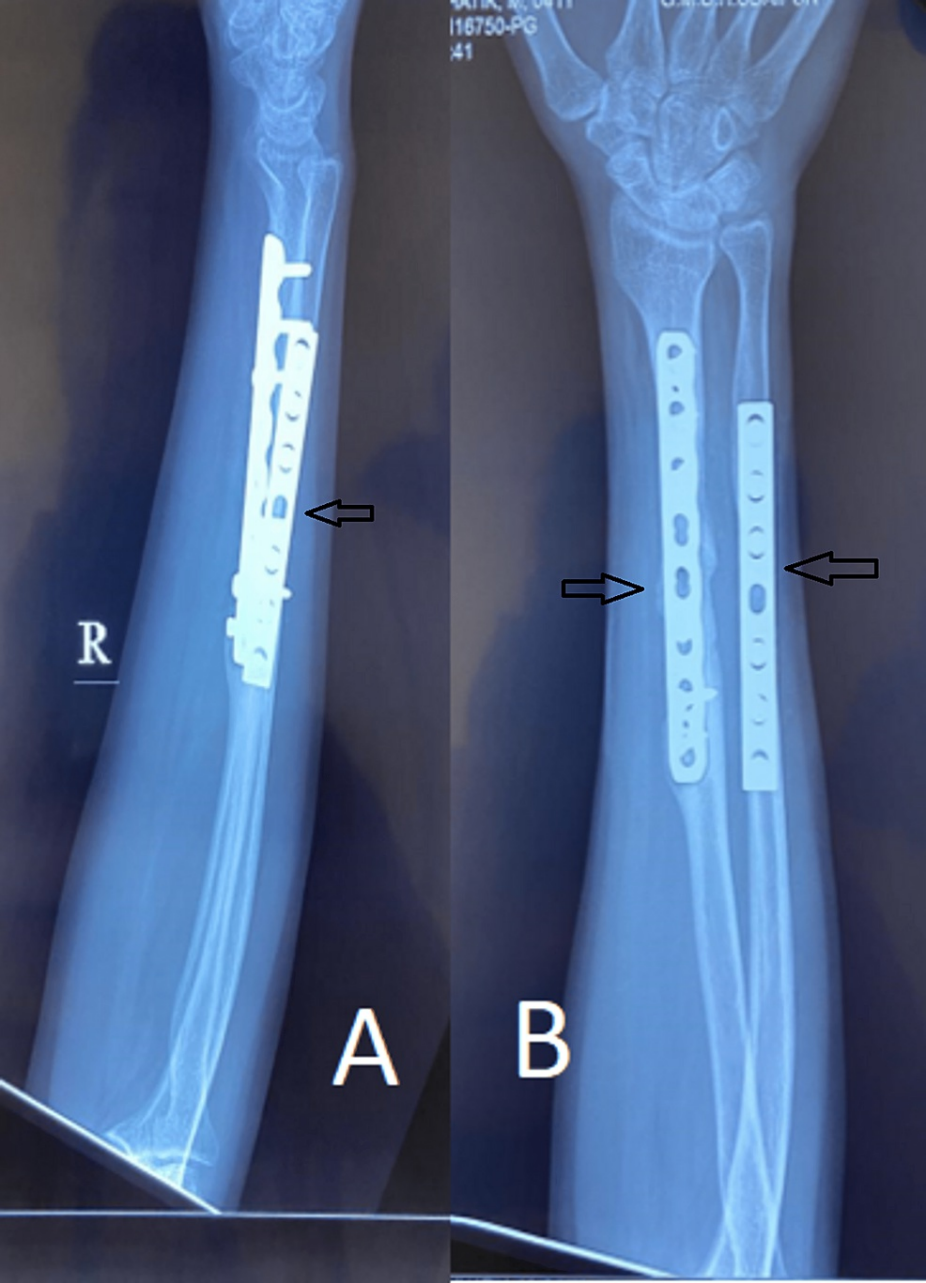
Sixth-month post-operative follow-up radiograph. Final follow-up radiograph showing fracture union (marked with arrows). Figure A is the lateral view, and figure B is the anteroposterior (AP) view.

**Figure 12:**
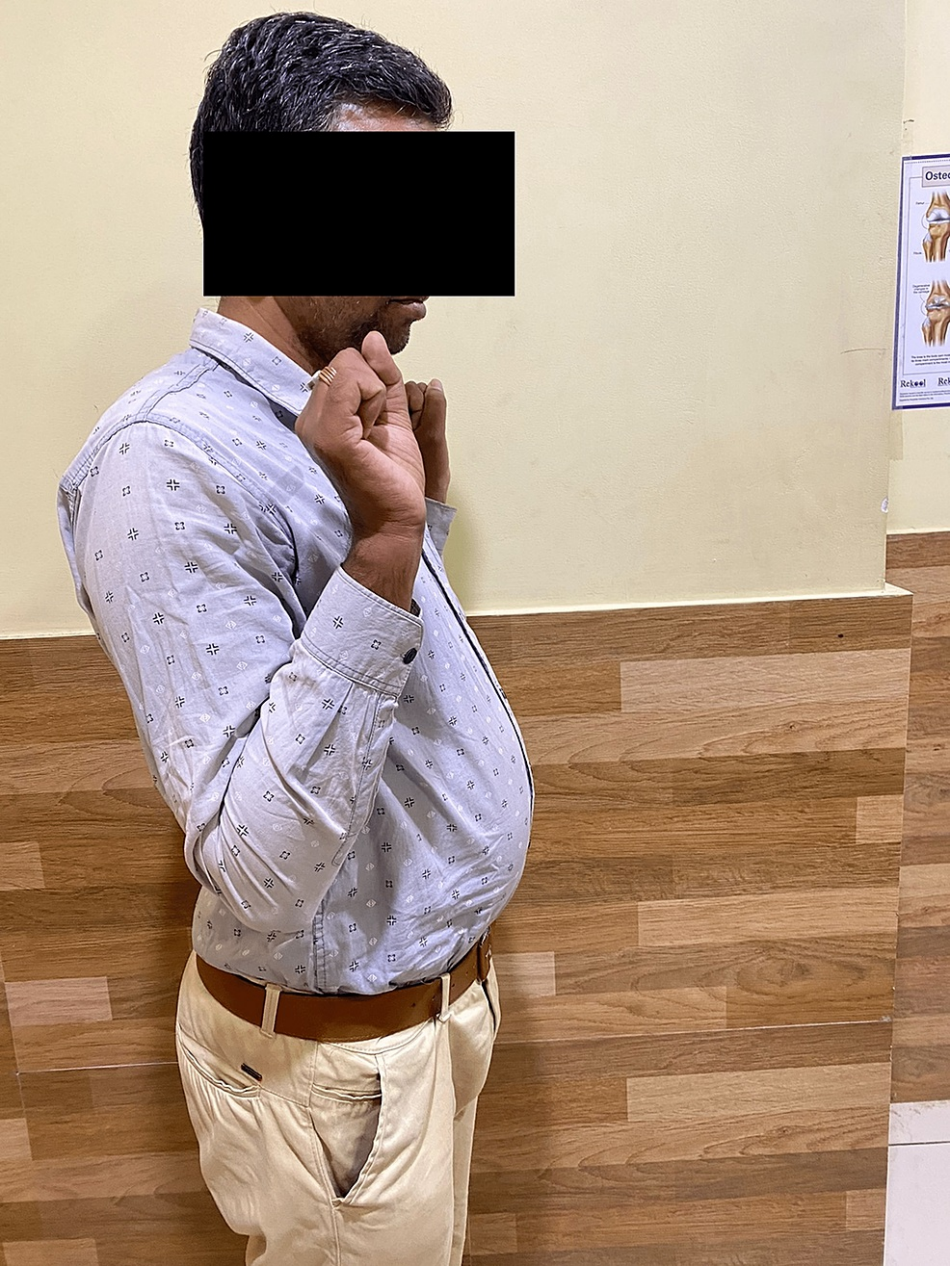
Clinical photograph at the final follow-up showing flexion movement at the elbow.

**Figure 13:**
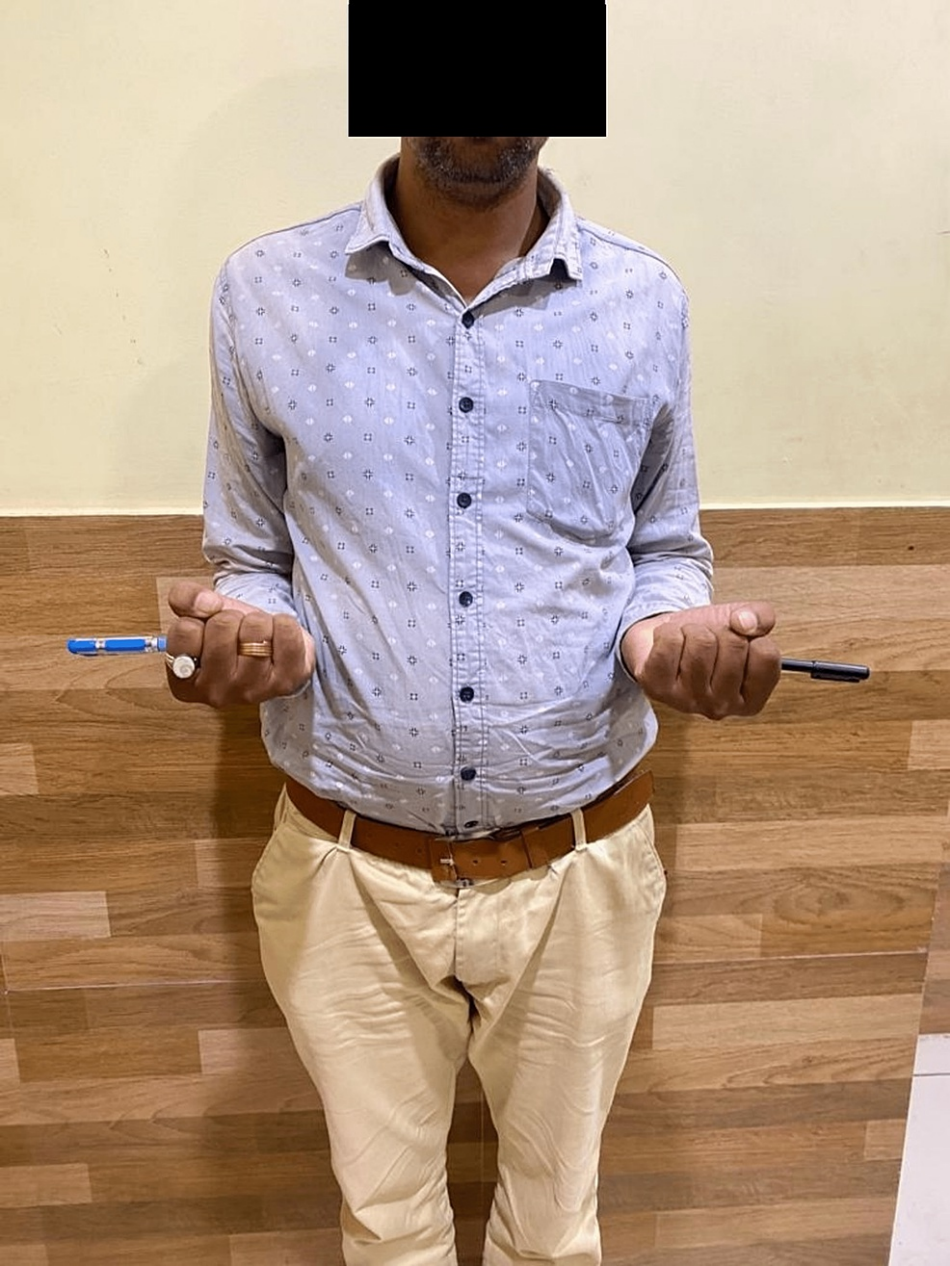
Clinical photograph showing supination movement at the elbow joint.

**Figure 14:**
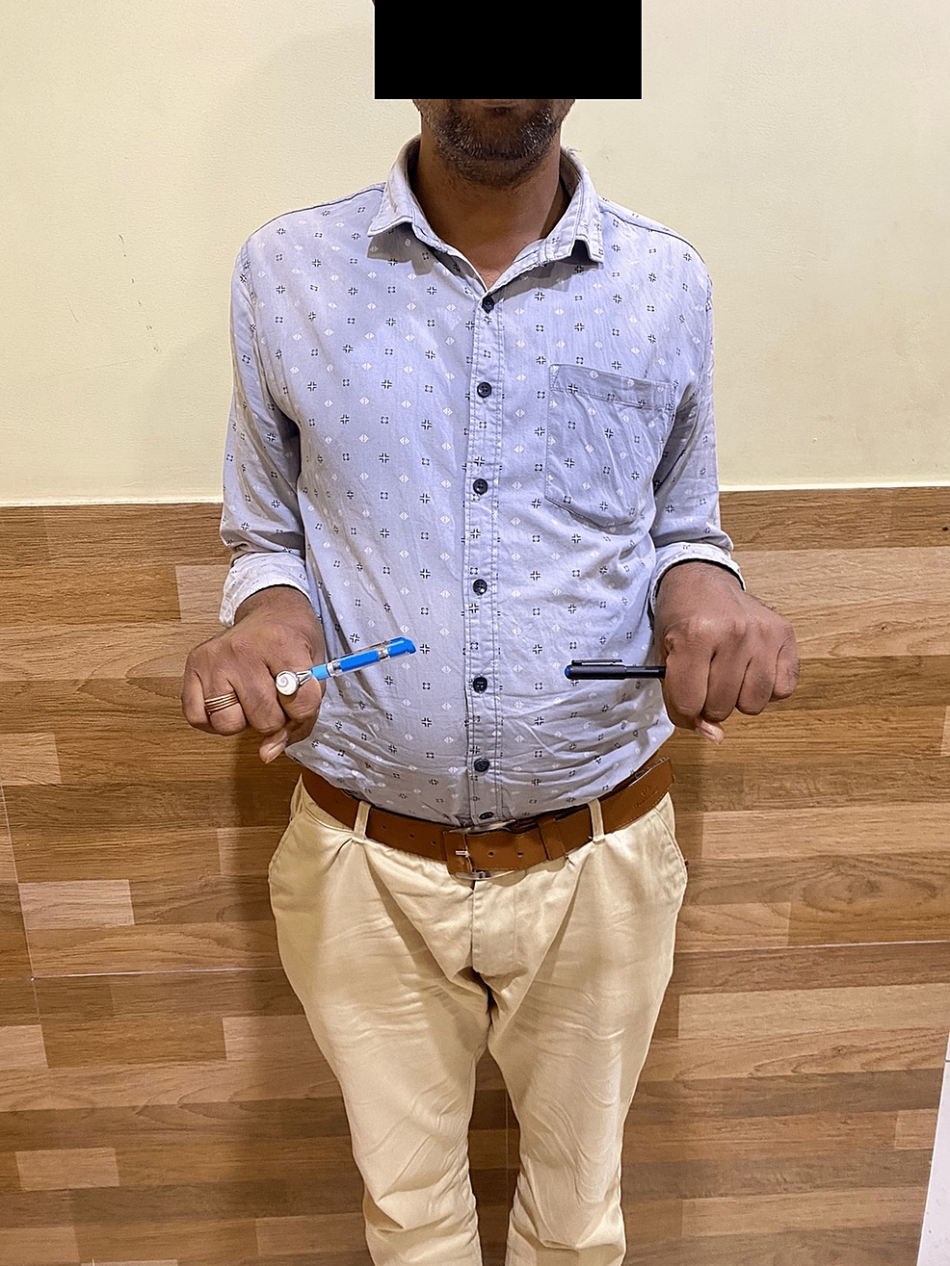
Clinical photograph showing pronation movement at the elbow joint.

Following are the radiographic images of one of our study cases managed by open reduction and internal fixation with small dynamic compression plates (DCP) (Figures 8, 9, 10, 11) and clinical images at the final follow-up (Figures 12, 13, 14).

## Discussion

Both bones of the forearm fracture are one of the most common fractures in adults in the upper extremity. Conservative treatment is associated with mal-union, resulting in decreased rotation of the forearm and poor outcomes. The loss of rotation impairs upper-limb function and daily activities.

For diaphyseal fractures of the forearm bones in adults, ORIF with dynamic compression plate (DCP) fixation has become the treatment of choice. The compression plating principle can be achieved by following the AO principles of internal fixation, which are anatomical fixation, preservation of vascularity, mechanically stable fixation, and rapid mobilization of adjacent joints. To achieve good results, adherence to AO principles, strict asepsis, proper post-operative rehabilitation, and patient education are required. As a result, excellent results from this technique of treatment have been documented in numerous studies. Closed intramedullary mechanical nails provide various advantages over the plate and screw fixation like they are minimally invasive procedure requiring shorter operating time [15].

In this study, we aimed to evaluate the clinical, radiological, and functional outcomes of diaphyseal fractures of both bones of the forearm in adult’s treated with plating and nailing.

The mean age of the patients in our study group who had adult diaphyseal fractures in both forearm bones was 36 years in group A (nailing) and 40 years in group B (plating), and 65% of the patients in both groups were between the ages of 31 and 50. Our findings were consistent with those of Michael et al. [16], Leung et al. [17], and Ravi et al. [18]. In our study group, men outnumbered women by a ratio of 80% to 20% in group A (nailing) and 90% to 10% in group B (plating). Our observation was in accordance with Ravi et al. [18] and Kumar [19]. In our study, the right side was more common in both groups. Ambhore et al. [2] and Polat et al. [20] studies also had a majority of fractures in the right forearm, which is correlated with our study. In this study, both groups were statistically similar in terms of age, gender, and side of injury.

We found that the most common cause of injury was a road traffic accident (RTA), which accounted for 47.5% of all patients. Other common causes of injury included falls in 32.5% of cases and assaults in 20% of cases. In comparison, studies conducted by Kumar [19], Moed et al. [21], and Grace et al. [22] found that in the majority of cases, RTA was the cause of injury, followed by assault, which is similar to our study.

In this study, we found that the average operative time for group B (plating) was 75.5 minutes, while group A (nailing) had a 57.25 minutes surgery. In terms of operating time, closed intramedullary nailing clearly has an advantage over the plating group. Smaller incisions, minimal soft tissue dissection, and preservation of vascularity at the fracture site have been described as advantages of nailing over plating. Our findings are consistent with the findings of Kumar [19] and Zhang et al [23].

In group A, two patients (10%) had surgical site infection (SSI), three patients (15%) had nail impingement, three cases (15%) had olecranon bursitis, one case (5%) had delayed union, and one case (5%) had non-union out of 20 cases. Whereas in group B, three cases (15%) had surgical site infection (SSI), three patients (15%) experienced elbow stiffness, and one patient (5%) had delayed union out of 20 cases. Patients with SSI and olecranon bursitis were successfully managed with regular dressings, antibiotics, and anti-inflammatory agents. Our study shows the same results as discussed in the literature; however, patients who had delayed union in group A and group B had shown union in later weeks of follow-up. One patient in the nailing group had non-union who was advised for plating with bone grafting, but the patient refused. The complications of studies conducted by Kumar [19], Al-Sadek et al. [24], Baldwin et al. [25], and Kim et al. [26] are comparable with our study. To summarize, it was discovered in our clinical study that patients in group B (plating) had fewer complications when compared to group A (nailing).

We observed that the mean time to union in patients who underwent plate osteosynthesis was 13.4 weeks, whereas in patients in the intramedullary nailing group, it was 10.4 weeks. In our study, the mean time to union in patients was consistent with the literature according to Kose et al. [27], Kibar et al. [28], and Cevik et al. [29]. We used a modified Grace and Eversman scoring system for evaluating the final functional results. In the nailing group (group A), out of 20 cases, excellent score was seen in six cases (30%), good in nine cases (45%), fair in three cases (15%), and at last, two cases (10%) showed poor results. In the plating group (group B), excellent scores were observed in eight cases (40%), good scores in eight cases (40%), fair scores in three cases (15%), and at last, one case (5%) had a poor outcome out of 20 cases. The causes for the poor outcomes were mainly late presentation to the hospital after an injury, poor compliance with physiotherapy protocol, and negligence.

## Conclusions

Among the two different surgical fixation modalities for diaphyseal fracture of both bone forearm in adults, the technique of open reduction and internal fixation (ORIF) with dynamic compression plate (DCP) exhibited good biomechanical stability, fewer complications and greater functional recovery than intramedullary nailing. Due to this, plate osteosynthesis appeared to be a superior alternative in clinical practice. However, smaller incisions, minimal soft tissue dissection, and preservation of vascularity at the fracture site are advantages of nailing over plating. We found that the complication rate was higher in the nailing group, but most of these complications were minor and easily manageable. Thus, we conclude that, as per our study for the treatment of diaphyseal fracture of both bones of the forearm, both treatment modalities provide equally satisfactory results (p-value 0.2014). However, the type of fracture, the effectiveness of the fracture reduction, and the choice of the operating surgeon could also have an impact on the clinical outcome.

## References

[1] Alffram PA, Bauer GC (1962). Epidemiology of fractures of the forearm: a biomechanical investigation of bone strength. J Bone Joint Surg Am.

[2] Ambhore N, Babhulkar S (2018). A comparative study between plating & intramedullary nailing for displaced diaphyseal fractures of radius and ulna in adults. Surg Rev.

[3] Ozkaya U, Kiliç A, Ozdoğan U, Beng K, Kabukçuoğlu Y (2009). Comparison between locked intramedullary nailing and plate osteosynthesis in the management of adult forearm fractures. Acta Orthop Traumatol Turc.

[4] Jónsson B, Bengnér U, Redlund-Johnell I, Johnell O (1999). Forearm fractures in Malmö, Sweden: changes in the incidence occurring during the 1950s, 1980s and 1990s. Acta Orthop Scand.

[5] Patrick J (1946). A study of supination and pronation, with especial reference to the treatment of forearm fractures. JBJS.

[6] Leung F, Chow SP (2006). Locking compression plate in the treatment of forearm fractures: a prospective study. J Orthop Surg (Hong Kong).

[7] Bucholz RW, Heckman JD, Court-Brown CM, Rockwood CA, Green DP (2006). Rockwood and Green's Fractures in Adults. https://www.worldcat.org/title/rockwood-and-greens-fractures-in-adults/oclc/300348277.

[8] Rao MR, Kader E, Sujith SV, Thomas V (2002). Nail-plate combination in management of fracture both bone forearm. J Bone Joint Surg.

[9] Dodge HS, Cady GW (19721). Treatment of fractures of the radius and ulna with compression plates: a retrospective study of one hundred and nineteen fractures in seventy-eight patients. JBJS.

[10] Muller M, Nazarian J, Koch P (1996). Fracture and dislocation compendium. Orthopaedic Trauma Association Committee for Coding and Classification. J Orthop Trauma.

[11] Gustilo RB, Anderson JT (1976). Prevention of infection in the treatment of one thousand and twenty-ﬁve open fractures of long bones: retrospective and prospective analyses. J Bone Joint Surg Am.

[12] Henry MH, Griggs SM, Levaro F, Clifton J, Masson MV (2001). Volar approach to dorsal displaced fractures of the distal radius. Tech Hand Upper Extremity Surg.

[13] Gadegone W, Salphale YS, Lokhande V (2012). Screw elastic intramedullary nail for the management of adult forearm fractures. Indian J Orthop.

[14] Grace TG, Eversmann WW Jr (1980). Forearm fractures: treatment by rigid fixation with early motion. J Bone Joint Surg Am.

[15] Schemitsch EH, Richards RR (1992). The effect of malunion on functional outcome after plate fixation of fractures of both bones of the forearm in adults. J Bone Joint Surg Am.

[16] Michael CW, Gordon JE, Zissimos AG (1989). Compression-plate fixation of acute fractures of the diaphyses of the radius and ulna. J Bone Joint Surg Am.

[17] Leung F, Chow SP (20031). A prospective, randomized trial comparing the limited contact dynamic compression plate with the point contact fixator for forearm fractures. JBJS.

[18] Ravi KB, Raghavendra TS, Balasubramanian S (2014). Forearm bone fractures: dynamic compression platting vs locking compression plating-randomised control study. Indian J Basic Appl Med Res.

[19] Kumar KH (2015). Study of different modalities of surgical treatment of diaphyseal fractures of forearm in adults. J Evol Med Dent Sci.

[20] Polat O, Toy S (2022). Comparison of the clinical and radiographic outcomes of plate fixation versus new-generation locked intramedullary nail in the management of adult forearm diaphyseal fractures. Acta Orthop Traumatol Turc.

[21] Moed BR, Kellam JF, Foster RJ, Tile M, Hansen Jr ST (1986). Immediate internal fixation of open fractures of the diaphysis of the forearm. JBJS.

[22] Grace TG, Eversmann WWJ (1980). Forearm fractures: treatment by rigid fixation with early motion. J Bone Joint Surg Am.

[23] Zhang XF, Huang JW, Mao HX, Chen WB, Luo Y (2016). Adult diaphyseal both-bone forearm fractures: a clinical and biomechanical comparison of four different fixations. Orthop Traumatol Surg Res.

[24] Al-Sadek TA, Niklev D, Al-Sadek A (2016). Diaphyseal fractures of the forearm in adults, plating or intramedullary nailing is a better option for the treatment?. Open Access Maced J Med Sci.

[25] Baldwin K, Morrison MJ 3rd, Tomlinson LA, Ramirez R, Flynn JM (2014). Both bone forearm fractures in children and adolescents, which fixation strategy is superior-plates or nails? A systematic review and meta-analysis of observational studies. J Orthop Trauma.

[26] Kim SB, Heo YM, Yi JW, Lee JB, Lim BG (2015). Shaft fractures of both forearm bones: the outcomes of surgical treatment with plating only and combined plating and intramedullary nailing. Clin Orthop Surg.

[27] Köse A, Aydın A, Ezirmik N, Yıldırım ÖS (2017). A comparison of the treatment results of dpen reduction internal fixation and intramedullary nailing in adult forearm diaphyseal fractures. Turk J Trauma Emerg Surg.

[28] Kibar B, Kurtulmuş T (2020). Comparison of new design locked intramedullary nails and plate osteosynthesis in adult isolated diaphyseal radius fractures. Eur J Trauma Emerg Surg.

[29] Çevik N, Akalin Y, Öztürk A (2020). The functional and radiological comparison of the surgical treatment results of forearm diaphyseal fractures in adults treated with open reduction internal fixation and intramedullary locking nail. Eur Res J.

